# Effect of 12-Week Intake of Nicotinamide Mononucleotide on Sleep Quality, Fatigue, and Physical Performance in Older Japanese Adults: A Randomized, Double-Blind Placebo-Controlled Study

**DOI:** 10.3390/nu14040755

**Published:** 2022-02-11

**Authors:** Mijin Kim, Jaehoon Seol, Toshiya Sato, Yuichiro Fukamizu, Takanobu Sakurai, Tomohiro Okura

**Affiliations:** 1 R&D Center for Tailor-Made QOL, University of Tsukuba, Tsukuba 305-8550, Japan; okura.tomohiro.gp@u.tsukuba.ac.jp; 2 Faculty of Health and Sport Sciences, University of Tsukuba, Tsukuba 305-8572, Japan; seol.jaehoon.ge@u.tsukuba.ac.jp; 3 Japan Society for the Promotion of Sciences, Tokyo 102-0083, Japan; 4 International Institute for Integrative Sleep Medicine (WPI-IIIS), University of Tsukuba, Tsukuba 305-8575, Japan; 5 Research and Development Division, Mitsubishi Corporation Life Sciences Limited, Tokyo 100-0006, Japan; toshiya.sato@mcls-ltd.com (T.S.); yuichiro.fukamizu@mcls-ltd.com (Y.F.); takanobu.sakurai@mcls-ltd.com (T.S.)

**Keywords:** nicotinamide mononucleotide, sleep quality, fatigue, physical performance, older adults

## Abstract

Deteriorating sleep quality and physical or mental fatigue in older adults leads to decreased quality of life and increased mortality rates. This study investigated the effects of the time-dependent intake of nicotinamide mononucleotide (NMN) on sleep quality, fatigue, and physical performance in older adults. This randomized, double-blind placebo-controlled study evaluated 108 participants divided into four groups (NMN_AM; antemeridian, NMN_PM; post meridian, Placebo_AM, Placebo_PM). NMN (250 mg) or placebo was administered once a day for 12 weeks. Sleep quality was evaluated using the Pittsburgh Sleep Quality Index. Fatigue was evaluated using the “Jikaku-sho shirabe” questionnaire. Grip strength, 5-times sit-to-stand (5-STS), timed up and go, and 5-m habitual walk were evaluated to assess the physical performance. Significant interactions were observed between 5-STS and drowsiness. 5-STS of all groups on post-intervention and drowsiness of the NMN_PM and Placebo_PM groups on mid- and post-intervention showed significant improvement compared with those in pre-intervention. The NMN_PM group demonstrated the largest effect size for 5-STS (d = 0.72) and drowsiness (d = 0.64). Overall, NMN intake in the afternoon effectively improved lower limb function and reduced drowsiness in older adults. These findings suggest the potential of NMN in preventing loss of physical performance and improving fatigue in older adults.

## 1. Introduction

The functions of human tissues and organs deteriorate gradually with aging, and cellular damage or stress also leads to cell senescence, which further contributes to dysfunction in various tissues [1]. In addition, oxidants produced by mitochondria accumulate alongside aging and cause oxidative damage, which further accelerates the aging of cells and tissues [2]. In particular, age-related mitochondrial DNA-deletion mutations accumulate focally in skeletal muscles, leading to fiber atrophy, loss of muscle mass, and dysfunction [3]. The decline in muscle and physical functions in the elderly may further cause geriatric syndromes, such as sarcopenia and frailty [4]. In addition, aging-induced insufficient physical activity and deterioration of physical function also result in fatigue. This symptom frequently occurs among the elderly and has been complained by 27–50% of community-dwelling older adults in their daily life. Fatigue is strongly associated with not only dullness and depression but also cognitive dysfunction and sleep disturbances [5,6]. In addition, chronic fatigue contributes to not only sleep disturbance and other sleep problems such as sleep latency, waking up during the night, waking up too early [7] but also physical dysfunction and physical inactivity. Aging is related to sleep disorders, and approximately 36.2% of older adults complain of insomnia [8]. The causes of sleep disorders are known as delayed circadian rhythm with aging [9,10], lower physical function and/or activity, and a decrease in social interaction [11]. Although hypnotic medications are used to solve these sleep disorders, they may cause side effects such as increased falls risk [12] and decreased cognitive functions [13]. Therefore, the American Geriatrics Society recommends that older adults avoid sleeping pills, as long-term dependence on medications such as sleeping pills can have adverse secondary health effects [14].

In particular, aggravation of physical or mental fatigue among the elderly further decreases physical activity and functions and increases the possibility of disease and mortality [15]. Since deterioration of health status in the elderly demonstrates a progressive change, from the appearance of risk factors, diseases, or impairments, functioning loss, disability, and death [16], effective interventions are of great necessity to improve their physical activity, fatigue, and sleep quality from an early stage.

Several nutritional intervention studies have demonstrated the effectiveness of various medicines and supplements for older adults, and nicotinamide mononucleotide (NMN) supplements have recently received particular attention. NMN is a precursor of nicotinamide adenine dinucleotide (NAD^+^) in the salvage pathway of NAD^+^ metabolism [17], and NAD^+^ is essential to maintain a healthy metabolism. It plays a critical role in the tricarboxylic acid cycle and is an important coenzyme in the intracellular redox reaction and electron carrier for all eukaryotes and a variety of archaea and eubacteria [18]. A decrease in intracellular NAD^+^ concentration has been implicated in the pathogenesis of age-related dysfunction in several tissues [19]. Reportedly, NAD^+^ levels can be increased by dietary nicotinamide riboside (NR) and NMN, the biosynthetic precursors of the salvage pathway in NAD^+^ metabolism [17], intake of NMN is expected to increase the concentration of NAD^+^. Studies have shown that NMN effectively activates the sirtuin genes related to aging, which may delay aging-induced physiological dysfunction [19], and Sirt1 is essential for maintaining physical activity, skeletal muscle mitochondrial function, and sleep quality [20]. Reportedly, NMN intake in mice effectively suppressed aging-related weight gain, enhanced energy metabolism, promoted physical activity, improved insulin sensitivity and plasma lipid profile, and ameliorated eye function [21].

These studies demonstrated the potential of NMN in activating mitochondrial function, preventing aging-induced diseases, and delaying the aging process in mice, indicating that NMN intake could benefit older adults by improving their physiological and psychological factors. To the best of the authors’ knowledge, the efficacy of NMN intake has only been reported in three human clinical trials [22,23,24]. However, these studies were conducted involving single-sex, small sample sizes, and non-older adults; therefore, limiting the generalization of the results of these studies to all populations. Considering these findings, this study hypothesized that NMN intake has a more positive effect on sleep quality, fatigue, and physical performance than placebo in older adults.

The present study was aimed to clarify the effects of the time-dependent intake of NMN for 12 weeks on sleep quality, fatigue, and physical performance in Japanese older adults using a randomized, double-blind, placebo-controlled parallel-group study.

## 2. Materials and Methods

### 2.1. Ethical Approval and Participants

This randomized, double-blind, placebo-controlled parallel-group study was approved by the ethical committees of the University of Tsukuba (reference no. Tai 019-24). The study protocol was registered at the University Hospital Medical Information Network center (UMIN no. 000038097). To ensure the reliability of this double-blind trial, the recruitment and data management of participants were entrusted to a business consignment agency (e-sports Co., Ltd., Tsukuba, Japan).

**Figure 1:**
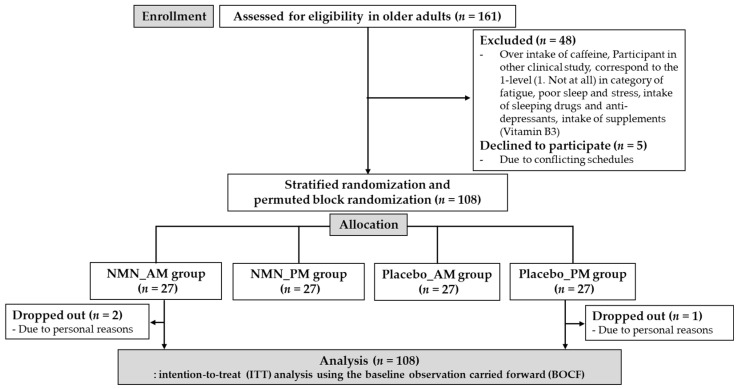
Flow-chart diagram.

Before collecting the sample for this study, the sample size was estimated using G*Power 3.1 analysis. The power analysis with an effect size = 0.25, α = 0.05, power (1 − β) = 0.95, number of groups = 4, and number of measurements = 3, identified the requirement of 60 participants as the ideal sample size. Participants were recruited through a regional information magazine (Joyo Living Co., Ltd., Tsukuba, Japan), and 161 older adults (≥65 years) residing in Tsukuba City, Japan, were included in this study. A screening survey via telephone and FAX was conducted using a questionnaire. The inclusion criteria were: (1) independent mobility and active participation in the study, (2) participants without dementia, and (3) no need for long-term care service. The participants were excluded if they (1) took sleeping drugs or anti-depressants regularly (more than once a week), (2) had an average intake of caffeine more than 400 mg per day, (3) took supplements or energy drinks containing NMN or niacin (Vitamin B3, nicotinamide, nicotinic acid amide), (4) had allergies to supplements, nutrition drinks or energy drinks containing NMN or niacin (Vitamin B3, nicotinamide, nicotinic acid amide), (5) had chronic fatigue syndrome, sleep disorders (insomnia), mental disorders such as depression or alcoholism and medical treatment, (6) corresponded to “1. Not at all” in the evaluation of fatigue, poor sleep, and stress on five levels [1. Not at all, 2. Slightly, 3. Moderately, 4. Very much, 5. Extremely], (7) had participated in other clinical experiments in the past 3 months, (8) were judged inappropriate by the lead principal investigator. Accordingly, based on the exclusion criteria, 48 participants were excluded. In addition, five participants declined to participate because of conflicting schedules. The study objective, design, inclusion and exclusion criteria, intervention of supplements, measurements, insurance compensation for injury, withdrawal of consent, and privacy protection were explained face-to-face to eligible participants. Finally, written consent to participate and data publishing was obtained from 108 participants (Figure 1).

### 2.2. Study Design

To compare the effect of intake at different times, NMN was supplemented in either the antemeridian (A.M.: after waking up until 12:00) or post meridian (P.M.: from 18:00 until bedtime). This study commissioned the e-sports Co., Ltd. to divide 108 participants into four groups using stratified randomization and permuted block randomization (NMN_AM, NMN_PM, Placebo_AM, Placebo_PM). First, this study randomly permuted the participants using random numbers. Second, participants were stratified by confounding factors including sex (male or female), age (young-old: 65–74 years or old-old: >75 years), and reporting of poor sleep (yes or no). Third, four permutation blocks were generated and randomly allocated to participants according to random numbers. To ensure the reliability of the double-blind study, all data (personal information, group information, distribution and collection of food and recording diary, and measurement results) were managed by e-sports Co., Ltd. The supplements for both the NMN and placebo groups were prepared to look identical. The e-sports Co., Ltd. distinguished the supplements for the four groups by ID numbers marked on top of the package and distributed them to the participants once every 3 weeks. All members remained completely anonymous to both participants and researchers until key codes were revealed after 12 weeks at the completion of this trial. During the 12-week intervention period (August to November 2019) conducted at the Innovation Medical Research Institute of the University of Tsukuba, primary outcomes, including sleep quality and fatigue, were investigated through subjective questionnaires at baseline (pre-intervention: Pre), the 6th week (mid-intervention: Mid), and the 13th week (post-intervention: Post), and physical performance was only evaluated at Pre and Post as the secondary outcome. Among the 108 participants, three dropped out due to personal reasons (Figure 1).

Participants were required to take six tablets of supplements (NMN or placebo, Mitsubishi Corporation Life Sciences Ltd., Tokyo, Japan) at once daily with water and to record their intake status (yes or no) for 12 weeks in the diary. These supplements were distributed to all participants once every 3 weeks; supplement intake was confirmed using self-reported diaries. Supplements in the NMN group consisted of NMN (250 mg), maltitol, crystalline cellulose, silicon dioxide, and magnesium stearate. Supplements in the placebo group consisted of maltitol, crystalline cellulose, silicon dioxide, and magnesium stearate.

### 2.3. Characteristics of Participants

Demographic and clinical characteristics were obtained using questionnaires and measurements. In brief, data on age, sex, medicine, medical history, smoking history, and alcohol consumption were obtained using a questionnaire. Subjective depressive symptoms were evaluated using the Japanese version of the Geriatric Depression Scale (GDS), a short version consisting of 15 items [25]. Body mass index (BMI, kg/m^2^) was calculated as body weight divided by height squared. Fat mass, muscle mass, and bone mass were measured using bioelectrical impedance analysis (BIA; MC-980A, TANITA, Tokyo, Japan). Systolic and diastolic blood pressure and heart rate were also measured (OMRON HEM-7111, Kyoto, Japan).

### 2.4. Sleep Quality

Subjective sleep quality was evaluated using the Pittsburgh Sleep Quality Index (PSQI) [26] and self-reported sleep diary. This study used the Japanese version of the PSQI, which has been proven as a reliable and valid tool in clinical practice and research [27]. With subscale scores ranging from 0 to 3, the PSQI assesses subjective sleep quality in the previous month and consists of seven items (sleep duration, sleep latency, sleeping medications, sleep disturbance, daytime dysfunction, sleep quality, and sleep efficiency). The total score of the PSQI, ranging from 0 to 21, is the sum of subscale scores, and the higher the scores, the lower the subjective quality of sleep.

### 2.5. Fatigue

Subjective fatigue was evaluated using the questionnaire “Jikaku-sho shirabe,” developed by the Industrial Fatigue Research Committee of Japan Occupational Health [28]. The questionnaire comprised 25 items divided into five categories—I: feeling of drowsiness (I feel drowsy, I feel a desire to lie down, I feel yawning, I feel a lack of a desire to do something, I feel tired in the whole body), II: feeling of instability (I feel anxious, I feel depressed, I feel restless, I feel nervous, I feel difficulty in thinking), III: feeling of uneasiness (I feel headache, I feel heavy in the head, I feel ill, I feel the brain hot or muddled, I feel dizziness), IV: feeling of local pain or dullness (I feel dullness in the arms, I feel lower back pain, I feel a pain in the hands or fingers, I feel tired in the legs, I feel tired in the neck and shoulders), V: feeling of eyestrain (I feel eye-blinking, I feel eyestrain, I feel pain in the eyes, I feel dry eyes, I feel blurred eyes). These feelings were evaluated on five levels from disagree completely to agree strongly with a score from 1 to 5 points (1: disagree completely, 2: agree scarcely, 3: agree slightly, 4: agree considerably, 5: agree strongly). We confirmed the reliability of all subordinate items of the Jikaku-sho shirabe by computing the Cronbach’s alpha coefficient (0.871), this result means that the data had acceptable internal consistency.

### 2.6. Physical Performance Tests

Physical performances were evaluated by grip strength, 5-times sit-to-stand (5-STS), timed up and go (TUG), and 5-m habitual walk. To measure grip strength (T.K.K.5401, Niigata, Japan), participants gripped a dynamometer with a maximum force twice on each hand in a standing position, and the average of the maximum value on each hand was used for analysis. For 5-STS, participants crossed their arms over chests and stood up and sat down on a chair as quickly as possible for five consecutive times. Participants performed the TUG, where they stood up from a chair, walked 3 m, turned around, returned, and sat on the chair as quickly as possible. To measure the 5-m habitual walk, participants were requested to walk through an 11-m straight course at normal speed, and the time between the 3-m and 8-m marks of the course was recorded. The 5-STS, TUG, and 5-m habitual walk were measured twice consecutively, and the fastest values were recorded as data. All measurement methods were based on previous studies [29].

### 2.7. Statistical Analyses

The primary outcomes were the changes in sleep quality and fatigue, and the secondary outcomes were changes in physical performance. Analysis of variance and chi-square test were used to compare the mean baseline characteristics among the four groups. Two-way repeated-measures analysis of variance was applied to evaluate differences in the effects of the intervention on sleep quality, fatigue, and physical performance. Based on the significant interactions (groups × times), post hoc analyses were conducted with Bonferroni corrections. This study used an intent-to-treat analysis to determine the effectiveness of the randomized controlled trials. In this study, three participants dropped out, and missing data were handled using the baseline observations carried forward. In addition, the effect sizes of all variables were evaluated by Cohen’s d value (small, d = 0.2; medium, d = 0.5; large, d = 0.8), where ‘d’ was defined as the difference between the means of Pre and Post (pre-post) divided by the pooled standard deviation (SD). The pooled SD is the root mean square of the SD of Pre and Post [30]. All statistical analyses were performed using SPSS version 26.0 (IBM Corp., Armonk, NY, USA) with the level of significance set at *p* < 0.05.

## 3. Results

### 3.1. Characteristics of Participants

No significant differences were observed in demographic and clinical variables among the four groups at baseline, and the intake rate of all groups was >95% (Table 1).

### 3.2. Sleep Quality

There was no statistically significant interaction in sleep quality as assessed by the PSQI. However, there were significant main effects (*p* < 0.01) of time in sleep duration, sleep disturbance score, daytime dysfunction score, sleep quality score, and total PSQI global score. In addition, the effect sizes of sleep latency (d = 0.56), daytime dysfunction score (d = 0.72), sleep quality score (d = 0.80), and total PSQI global score (d = 0.68) in the NMN_PM group were the largest among the four groups (Table 2).

### 3.3. Fatigue

Table 3 shows the results of the fatigue. The interactions of fatigue with drowsiness were significant (*p* = 0.02). According to the post hoc analysis, the improvement in the drowsiness of Mid and Post was more significant than that of Pre in both NMN_PM and Placebo_PM groups. Drowsiness (*p* < 0.01), instability (*p* < 0.01), dullness (*p* = 0.03), and total fatigue score (*p* < 0.01) showed significant in the main effects of time. In addition, the NMN_PM group showed a medium effect size (d = 0.64) in drowsiness, and it was the largest among the groups.

### 3.4. Physical Performances

As shown in Table 4, a significant interaction was observed in 5-STS (*p* = 0.04). The post hoc analysis revealed significantly improved 5-STS in Post compared with Pre in all groups. Significant main effects of time were observed in 5-STS (*p* < 0.01) and TUG (*p* < 0.01). Further, 5-STS showed significant in the main effects of group (*p* = 0.05). In addition, the effect size of 5-STS was the largest in the NMN_PM group (d = 0.72) compared with the other three groups. Furthermore, the NMN_PM group showed a medium effect size (d = 0.54) in TUG, which was the largest among the groups.

## 4. Discussion

An increase in NAD^+^ through NMN ingestion is expected to improve aging-related mitochondrial disorders, chronic inflammation, oxidative stress, DNA damage, and sirtuin inactivation [31,32], which may prevent the onset of age-related diseases and physical dysfunction. For example, a previous study reported that NMN intake (100 mg/kg/day) significantly increased ambulation (whole-body movements) in mice when compared with the control group [21]. Similarly, the present study demonstrated that NMN intake in the afternoon was the most effective in enhancing the functions of the lower extremities evaluated by 5-STS and TUG when compared to other groups, which provides supporting evidence for previous studies and indicates that NMN intake is effective in improving mobility in both mice and humans. Although previous cellular, animal, and human clinical studies have investigated the efficacy of NMN intake on aging-related changes in functions and diseases, only three studies have verified the effect of NMN intake on humans, which is far from sufficient to verify the effect of NMN intake in older adults.

Among the three human clinical studies, the first study was a randomized, double-blind, placebo-controlled trial [24]. In this study, NMN (300 mg, 600 mg, 1200 mg) or placebo was administered daily with exercise to 48 young runners. Both medium- and high-dose groups showed a significant improvement in aerobic capacity, which was inferred to result from improved O_2_ utilization in skeletal muscle [24]. Despite the difference in study design between the previous study and this study, NMN was shown to improve aerobic capacity in humans, which is promising for further improvement of physical functions. The second human clinical study investigated the effects of NMN intake (250 mg/day) in obese postmenopausal women with prediabetes (NMN = 13, placebo = 12) for 10 weeks in a randomized, placebo-controlled, double-blind trial, reporting that significant improvement was found in protein kinase AKT, mTOR protein, and insulin sensitivity, whereas no significant changes were found in grip strength, fatigability, and recovery from the fatiguing exercise of the leg [23]. On the contrary, in a cross-sectional study, daily intake of dietary nicotinamide, which has a different salvage pathway from NMN, was reported to be significantly associated with not only higher grip strength but also lower subjective fatigue in colorectal cancer survivors [33]. In this study, despite the non-significant change in grip strength, NMN intake in the afternoon was effective in improving drowsiness among several factors of subjective fatigue. Chronic fatigue is more likely to occur among older adults because of their poor ability to recover from fatigue; moreover, older adults are the majority to complain about fatigue induced by aging and diseases. Therefore, it can be inferred that improving fatigue through NMN supplementation could be meaningful for older adults.

To the best of the authors’ knowledge, this study is the first to demonstrate the effects of the time-dependent intake of NMN on older adults. Fatigue usually occurs more frequently in the afternoon when most daily activities end in a day, and severe fatigue may negatively influence sleep quality without complete recovery. In severe situations, sleep disorders may further contribute to daytime sleepiness or fatigue on the following day [34]. Therefore, examination of the effects of the time-dependent intake of NMN was considered necessary, and two time zones (morning and afternoon) were set to investigate the optimal intake time to achieve the maximum efficiency of NMN absorption. Although no human clinical study has reported that time-dependent intake of NMN leads to differences in its effects, activation of NAD^+^ has been reported to vary with time after NMN intake in mice [21]. In brief, the study of Mills et al. has shown that after NMN ingestion, NMN in plasma increased rapidly at 2.5 min and began to be converted into NAD^+^ from 10 min, accompanied by a significant increase in NAD^+^ from 30 min [21]. These results suggested that a certain amount of time is required to activate NAD^+^ after NMN intake. In this study, only the NMN_PM group, the group that ingested NMN after 18:00, showed significant improvement in sleep quality with a significant reduction in drowsiness, which may further improve physical performance. The intake of milk containing 10–40 ng/L melatonin at night has been reported to significantly improve sleep quality and daily activities in elderly adults compared to the intake in the daytime [35], illustrating that supplementation ingested in the afternoon, rather than morning, seems more effective in improving sleep quality. In addition, although its effect in humans is unknown, co-administration of melatonin (10 mg/kg) and NMN (100 mg/kg) to mice has been shown to alleviate age-related memory impairment and reduce mitochondrial dysfunction as well as apoptotic cells in both prefrontal cortex and hippocampus regions [36].

In terms of the third human clinical study, safety and pharmacokinetics of NMN were verified in healthy Japanese men (*n* = 10) by a single-arm intervention trial, reporting no significant change in sleep quality, which was investigated by PSQI as this study did, before and after intervention [22]. In this study, no significant interaction between groups and time was observed; however, the NMN_PM group showed the largest effect sizes in sleep latency, daytime dysfunction score, sleep quality score, and total PSQI global score compared to the other groups. Although no previous studies have directly examined the mechanism underlying the effect of NMN or NAD^+^ on sleep-related factors, sirtuins are enzymes dependent on NAD^+^ to deacetylate proteins, and Sirt1-dependent neural activation in the dorsomedial hypothalamic nucleus and lateral hypothalamus have been reported to prevent the aging-induced decline in skeletal muscle mitochondrial function, physical activity, body temperature, oxygen consumption, and quality of sleep in mice [20]. Despite the relatively weak explanatory power of sleep quality evaluated by a subjective questionnaire, this study suggested that NMN intake may have a positive effect on sleep parameters, as reported in previous studies [20]. However, it is necessary to further verify the effect of NMN intake on sleep, as evaluated by objective measures in the future. 

Considering the dose-dependent aspect of NMN, a previous study has compared the effects of oral NMN intake of 100, 250, and 500 mg [22], whereas, in other studies, NMN at 250 mg/day [23], 300 mg/day, 600 mg/day, and 1200 mg/day was administered [24]. In this study, the NMN group took 250 mg/day every day for 12 weeks, and the intake rate of supplementation was more than 95%. In addition, compared to previous studies, this study does not seem to have any quantitative problems, and there were no reported side effects from NMN ingestion.

This study has several limitations. First, a daily diet that may contain NNM was not controlled, and a survey on daily nutrient intake was not conducted. Although a previous study has reported that NMN is also present in foods such as vegetables, fruits, meat, and seafood, the amount of NMN in daily food is quite small, i.e., less than 1.8 mg per 100 g of food consumed [21]. Thus, it remains unclear whether the daily diet of the participants affected the effectiveness of NMN. In future studies, it will be necessary to conduct a survey on nutrient intake in daily life and additional analyses to examine the double effect. Second, changes in physiological factors that could have been caused by NMN intake were not examined. Here, the effect of NMN intake was examined only through physical measurements and questionnaires. In contrast, the direct effect of NMN on physiological factors in vivo remains unclear, which could be ascertained by examination of the physiological changes in blood, urine, and muscle in the future. In addition, it is necessary to add evaluation such as actigraphy, polysomnography, and sleep diary in future research. Third, a placebo effect on 5-STS, drowsiness, and sleep disturbance scores was observed in this study. Although researchers and participants had been kept double-blind until the experiment finished, as we had to explain the study design to participants under research ethics guidelines during the research briefing, participants might have perceived the placebo as NMN, which could be the reason for the observed placebo effects and the non-significant interaction between the four groups. Recently, NMN supplements for anti-aging have been launched on the market; however, the authors of this study still hold the opinion that the effectiveness and safety of NMN should be clarified through further human clinical studies.

## 5. Conclusions

This study showed a significant interaction between 5-STS and drowsiness after NMN intake for 12 weeks. The post hoc test and effect size revealed that 5-STS and drowsiness were significantly improved in the NMN_PM group. Collectively, this study suggests that NMN intake in the afternoon is more effective in improving lower limb function and reducing drowsiness in older adults, which could further benefit their physical and mental health.

## Figures and Tables

**Table 1:** Comparison among characteristics of participants in four groups.

Variables (Unit)	NMN_AM (*n* = 27)			NMN_PM (*n* = 27)			Placebo_AM (*n* = 27)			Placebo_PM (*n* = 27)			*p*-Value
	Mean	±	SD	Mean	±	SD	Mean	±	SD	Mean	±	SD	
Age (year)	72.2	±	5.1	72.8	±	4.3	72.5	±	4.6	73.0	±	4.7	0.92
BMI (kg/m^2^)	22.9	±	2.5	23.4	±	2.8	22.3	±	3.2	22.4	±	2.2	0.46
Systolic blood pressure (mmHg)	141.0	±	14.5	140.3	±	15.0	139.0	±	15.4	137.2	±	16.8	0.82
Diastolic blood pressure (mmHg)	77.2	±	7.8	79.3	±	12.9	75.7	±	10.4	72.9	±	8.4	0.13
Heart rate (bpm)	76.4	±	12.2	78.1	±	10.2	77.1	±	9.9	76.4	±	13.5	0.94
Fat mass (kg)	15.2	±	5.3	15.6	±	6.3	14.3	±	5.5	14.7	±	5.6	0.86
Muscle mass (kg)	38.0	±	6.6	38.9	±	7.2	37.7	±	7.7	37.3	±	6.4	0.86
Bone mass (kg)	2.2	±	0.4	2.2	±	0.4	2.1	±	0.4	2.1	±	0.3	0.80
GDS score (point)	1.6	±	2.1	1.5	±	1.8	1.0	±	1.3	1.1	±	1.4	0.58
^†^ Women (*n* (%))	18 (66.7)			18 (66.7)			18 (66.7)			19 (70.4)			0.99
^†^ Poor sleep (yes; *n* (%))	19 (70.4)			15 (55.6)			12 (44.4)			12 (44.4)			0.18
^†^ Medicine (yes; *n* (%))	16 (59.3)			15 (55.6)			16 (59.3)			19 (70.4)			0.70
^†^ Medical history (yes; *n* (%))	22 (81.5)			22 (81.5)			23 (85.2)			24 (88.9)			0.86
^†^ No-smoking (*n* (%))	21 (77.8)			21 (77.8)			19 (70.4)			20 (74.1)			0.89
^†^ Quit smoking (*n* (%))	6 (23.1)			6 (22.2)			7 (25.9)			6 (22.2)			
^†^ Smoking (*n* (%))	0			0			1 (3.7)			1 (3.7)			
^†^ No-drinking (*n* (%))	17 (63.0)			14 (51.9)			12 (44.4)			13 (48.1)			0.66
^†^ ≤Drinking one day a month (*n* (%))	0			3 (11.1)			2 (7.4)			1 (3.7)			
^†^ Drinking 2~3 days a month (*n* (%))	4 (14.8)			2 (7.4)			5 (18.5)			3 (11.1)			
^†^ ≤Drinking 1~7 days a week (*n* (%))	6 (22.2)			8 (29.6)			8 (29.6)			10 (37.0)			
^†^ Intake rate of supplementation (%)	95.7	±	7.8	97.8	±	3.3	98.5	±	3.0	98.2	±	2.5	0.12

Note: Analysis of variance, ^†^: Chi-square test, *n*: number of participants, SD: standard deviation, NMN: nicotinamide mononucleotide, BMI: body mass index, GDS: geriatric depression scale.

**Table 2:** Effect of supplementation on sleep quality in four groups.

Variables (Unit)	Time	NMN_AM ^A^			NMN_PM ^B^			Placebo_AM ^C^			Placebo_PM ^D^			Main Effect of Group *p*-Value	Main Effect of Time *p*-Value	Interaction *p*-Value (Groups × Times)
		Mean	±	SD	Mean	±	SD	Mean	±	SD	Mean	±	SD			
Sleep duration (hour)	Pre	6.3	±	1.1	6.1	±	1.1	6.5	±	0.9	6.4	±	1.2	0.81	<0.01	0.72
	Mid	6.4	±	1.1	6.4	±	1.4	6.5	±	0.9	6.5	±	0.9			
	Post	6.4	±	1.2	6.5	±	1.1	6.7	±	0.9	6.7	±	1.0			
	d	0.14			0.34			0.25			0.31					
Sleep latency (minute)	Pre	22.9	±	24.4	19.8	±	17.1	23.9	±	19.0	23.7	±	29.2	0.44	0.08	0.23
	Mid	34.6	±	71.5	16.3	±	13.2	16.0	±	12.5	16.5	±	14.2			
	Post	16.5	±	16.5	12.0	±	9.7	15.5	±	13.1	17.6	±	22.6			
	d	0.31			0.56			0.52			0.23					
Sleeping medication score (point)	Pre	1.0	±	0.2	1.1	±	0.4	1.1	±	0.3	1.0	±	0	0.60	0.24	0.53
	Mid	1.1	±	0.3	1.0	±	0	1.1	±	0.4	1.0	±	0			
	Post	1.0	±	0.2	1.0	±	0	1.0	±	0	1.0	±	0			
	d	―			0.27			0.39			―					
Sleep disturbance score (point)	Pre	7.7	±	4.8	6.6	±	3.3	6.1	±	4.1	7.4	±	4.1	0.18	<0.01	0.81
	Mid	6.9	±	4.4	5.3	±	4.4	5.1	±	3.7	5.1	±	3.4			
	Post	6.6	±	3.2	4.9	±	2.8	4.4	±	3.5	4.9	±	3.9			
	d	0.29			0.55			0.47			0.61					
Daytime dysfunction score (point)	Pre	1.9	±	1.3	1.7	±	1.1	1.5	±	1.2	1.0	±	0.9	0.07	<0.01	0.08
	Mid	1.1	±	0.9	1.1	±	1.1	1.1	±	0.9	1.0	±	1.1			
	Post	1.5	±	1.4	1.0	±	0.9	0.9	±	0.8	0.6	±	0.9			
	d	0.27			0.72			0.61			0.38					
Sleep quality score (point)	Pre	2.5	±	0.6	2.4	±	0.6	2.3	±	0.7	2.4	±	0.5	0.38	<0.01	0.42
	Mid	2.3	±	0.5	2.2	±	0.6	2.1	±	0.7	2.1	±	0.4			
	Post	2.3	±	0.7	2.0	±	0.4	2.1	±	0.6	2.0	±	0.4			
	d	0.24			0.80			0.22			0.72					
Sleep efficiency (%)	Pre	87.0	±	13.5	92.3	±	12.3	94.4	±	22.8	88.2	±	16.7	0.49	0.68	0.23
	Mid	88.0	±	15.0	90.2	±	14.2	88.5	±	9.1	90.9	±	15.0			
	Post	85.2	±	14.6	91.1	±	13.3	91.4	±	8.4	91.8	±	17.1			
	d	0.12			0.10			0.18			0.21					
Total PSQI global score (point)	Pre	7.2	±	3.1	6.7	±	2.3	6.2	±	2.6	6.3	±	3.3	0.26	<0.01	0.87
	Mid	6.5	±	3.0	5.7	±	2.2	5.6	±	2.4	5.3	±	2.6			
	Post	6.3	±	3.2	5.2	±	2.1	5.0	±	2.0	4.7	±	2.7			
	d	0.31			0.68			0.51			0.52					

Note: Two-way repeated-measures analysis of variance, d: effect size (Cohen’s d), Pre vs. Post (0.2: small, 0.5: medium, 0.8: large), PSQI: Pittsburgh Sleep Quality Index. ^A^: NMN_AM, ^B^: NMN_PM, ^C^: Placebo_AM, ^D^: Placebo_PM.

**Table 3:** Effect of supplementation on fatigue in four groups.

Variables (Unit)	Time	NMN_AM ^A^			NMN_PM ^B^			Placebo_AM ^C^			Placebo_PM ^D^			Main Effect of Group *p*-Value	Main Effect of Time *p*-Value	Interaction *p*-Value (Groups × Times)	Post Hoc Analysis with Bonferroni Correction
		Mean	±	SD	Mean	±	SD	Mean	±	SD	Mean	±	SD				
Drowsiness (point)	Pre	^§^ 9.7	±	3.8	10.0	±	3.5	8.5	±	2.9	9.1	±	3.5	0.05	<0.01	0.02	^B, D^: Mid, Post < Pre Mid: ^D^ < ^A^
	Mid	^§^ 10.3	±	3.9	8.3	±	2.9	8.6	±	2.6	7.1	±	2.5				
	Post	^§^ 9.4	±	3.8	7.9	±	2.9	7.5	±	2.5	7.5	±	2.6				
	d	0.08			0.64			0.37			0.50						
Instability (point)	Pre	8.1	±	3.1	8.4	±	3.0	8.4	±	2.9	8.1	±	3.9	0.70	<0.01	0.33	
	Mid	8.5	±	3.4	7.3	±	2.9	7.2	±	1.8	7.0	±	2.8				
	Post	7.8	±	2.7	7.6	±	2.6	7.5	±	2.3	6.9	±	2.8				
	d	0.09			0.29			0.34			0.36						
Uneasiness (point)	Pre	7.1	±	2.1	7.1	±	2.4	7.4	±	2.3	7.3	±	3.0	0.64	0.10	0.47	
	Mid	7.6	±	2.8	6.5	±	1.8	7.0	±	2.3	6.5	±	2.5				
	Post	7.0	±	1.9	6.6	±	2.5	7.0	±	2.3	6.3	±	2.0				
	d	0.04			0.21			0.16			0.37						
Dullness (point)	Pre	9.7	±	3.7	10.1	±	4.3	10.4	±	3.7	10.2	±	3.4	0.88	0.03	0.35	
	Mid	10.1	±	3.8	9.1	±	4.0	9.8	±	2.9	8.9	±	3.0				
	Post	9.2	±	3.5	9.2	±	5.0	10.1	±	4.1	9.2	±	3.1				
	d	0.14			0.18			0.09			0.31						
Eyestrain (point)	Pre	10.4	±	4.6	10.1	±	4.9	10.0	±	4.3	10.1	±	3.9	0.86	0.27	0.58	
	Mid	10.7	±	5.1	9.9	±	4.8	9.6	±	4.3	9.6	±	4.0				
	Post	10.0	±	4.7	10.4	±	5.2	8.9	±	3.5	9.5	±	3.6				
	d	0.10			0.06			0.30			0.15						
Total fatigue score (point)	Pre	^§^ 45.3	±	15.1	45.7	±	15.5	44.8	±	12.5	44.8	±	14.5	0.64	<0.01	0.19	
	Mid	^§^ 47.3	±	16.3	41.1	±	12.6	42.3	±	9.6	39.0	±	12.5				
	Post	^§^ 43.5	±	13.0	41.8	±	15.7	41.0	±	11.6	39.5	±	11.2				
	d	0.13			0.25			0.31			0.41						

Note: Two-way repeated-measures analysis of variance, d: effect size (Cohen’s d), Pre vs. Post (0.2: small, 0.5: medium, 0.8: large), ^§^: 26 participants. Higher points of all variables mean worse fatigue. ^A^: NMN_AM, ^B^: NMN_PM, ^C^: Placebo_AM, ^D^: Placebo_PM.

**Table 4:** Effect of supplementation on physical performances in four groups.

Variables (Unit)	Time	NMN_AM ^A^			NMN_PM ^B^			Placebo_AM ^C^			Placebo_PM ^D^			Main Effect of Group *p*-Value	Main Effect of Time *p*-Value	Interaction *p*-Value (Groups × Times)	Post Hoc Analysis with Bonferroni Correction
		Mean	±	SD	Mean	±	SD	Mean	±	SD	Mean	±	SD				
Grip strength (kg)	Pre	^§^ 27.9	±	6.4	26.9	±	8.1	27.4	±	6.0	26.6	±	6.5	0.84	0.09	0.78	
	Post	^§^ 28.6	±	7.0	27.2	±	6.8	27.5	±	6.4	26.8	±	6.3				
	d	0.10			0.05			0.02			0.03						
^†^ 5-times sit to stand (s)	Pre	^§^ 5.5	±	1.1	6.3	±	1.4	5.6	±	0.8	6.4	±	1.7	0.05	<0.01	0.04	^A, B, C, D^: Post < Pre
	Post	^§^ 5.1	±	0.7	5.3	±	1.1	5.3	±	1.0	5.9	±	1.7				
	d	0.40			0.72			0.41			0.30						
^†^ Timed up and go (s)	Pre	5.4	±	0.8	5.5	±	0.6	5.2	±	0.6	5.6	±	1.3	0.30	<0.01	0.36	
	Post	5.2	±	0.8	5.2	±	0.6	5.0	±	0.4	5.4	±	1.2				
	d	0.22			0.54			0.36			0.20						
^†^ 5-m habitual walk (s)	Pre	3.3	±	0.5	3.3	±	0.6	3.2	±	0.4	3.4	±	0.7	0.42	0.47	0.26	
	Post	3.4	±	0.5	3.4	±	0.6	3.2	±	0.4	3.4	±	0.7				
	d	0.12			0.22			0.06			0.09						

Note: Two-way repeated-measures analysis of variance, d: effect size (Cohen’s d), Pre vs. Post (0.2: small, 0.5: medium, 0.8: large), ^§^: 26 participants. ^†^: Lower values mean faster performance. ^A^: NMN_AM, ^B^: NMN_PM, ^C^: Placebo_AM, ^D^: Placebo_PM.

## Data Availability

All data generated or analyzed during this study are included in this published article. In addition, upon reasonable request, data supporting the findings of the study are provided by the corresponding author.
